# Common fragile sites: protection and repair

**DOI:** 10.1186/s13578-020-00392-5

**Published:** 2020-03-05

**Authors:** Shibo Li, Xiaohua Wu

**Affiliations:** Department of Molecular Medicine, The Scripps Research Institute, La Jolla, San Diego, CA 92037 USA

**Keywords:** Common fragile sites, Replication stress, DNA secondary structures, AT-rich sequences, Break-induced replication

## Abstract

Common fragile sites (CFSs) are large chromosomal regions that exhibit breakage on metaphase chromosomes upon replication stress. They become preferentially unstable at the early stage of cancer development and are hotspots for chromosomal rearrangements in cancers. Increasing evidence has highlighted the complexity underlying the instability of CFSs, and a combination of multiple mechanisms is believed to cause CFS fragility. We will review recent advancements in our understanding of the molecular mechanisms underlying the maintenance of CFS stability and the relevance of CFSs to cancer-associated genome instability. We will emphasize the contribution of the structure-prone AT-rich sequences to CFS instability, which is in line with the recent genome-wide study showing that structure-forming repeat sequences are principal sites of replication stress.

## Background

Common fragile sites (CFSs) are normal chromosomal regions that recurrently form cytogenetically defined gaps and breaks on metaphase chromosomes upon partial inhibition of DNA synthesis [1]. Prominently, CFSs are hotspots for chromosomal instability and rearrangements in cancers. They are often associated with deletions of tumor suppressor genes and amplification of oncogenes [2–5], and are highly prone to the occurrence of copy number variation (CNV) [6]. They are also preferred sites for viral integration which could lead to cancer development [7–10]. Since CFS instability occurs in the pre-cancerous stage, preceding the instability at other genome loci [11–14], genome instability at CFSs is thought to be a driving force for tumorigenesis.

It has long been known that CFSs exhibit multiple characteristics which contribute to their fragility. CFSs contain difficult-to-replicate DNA sequences such as AT-rich sequences, which tend to form DNA secondary structures to stall DNA replication [15–19]. CFSs are often replicated late [20, 21] and also have a shortage of replication origins [22–25]. They often contain very large genes, which cause conflicts between replication and transcription [26]. Although these features disturb replication progress at CFSs under normal conditions, CFSs are still well maintained and are stable in general. However, upon replication stress, replication at CFSs is disturbed and further delayed, which then leads to incomplete DNA replication of CFSs when cells enter mitosis, resulting in CFS expression (a tterm to describe CFS breakage on metaphase chromosomes) [27, 28]. It is well accepted that CFS expression is not simply caused by a single feature of CFSs, but rather by a combination of more than one mechanism. For instance, replication is often stalled in CFSs due to secondary structure formation at AT-rich sequences or conflict between active transcription and replication, while CFSs are scarce in replication origins that are needed to timely complete DNA replication. The combination of fork stalling and the paucity of replication origins leads to CFS expression. Growing evidence shows that CFS instability varies among distinct cell types as well as in response to different growth conditions, suggesting that the maintenance of genome stability at CFSs has a complex nature [29, 30]. On the other hand, it has also been well established that all CFSs share a unique common feature that they are all sensitive to replication stress. In this review, we will focus on discussing the mechanisms that underlie the protection of CFSs from chromosomal breakage and the repair of CFSs once they are broken under replication stress.

### Basic features of CFSs

The expression of CFS on metaphase chromosomes suggests that these regions either fail to complete DNA replication in the S-phase and G2-phase or suffer breakages that are carried over to mitosis. Several features of CFSs when combined together cause CFS expression. First, late replication timing is one recognizable feature of CFSs. For instance, replication of FRA3B occurs very late in unperturbed cells, and more than 10% of FRA3B remains unreplicated in G2 after aphidicolin (APH) treatment [20]. FRA16D also replicates in late S-phase [31]. In some other CFSs, replication starts in early to middle S-phase, but exhibits a significant delay in replication progression, resulting in incomplete replication of large regions of these CFSs [3, 21, 32]. However, late replication alone is not sufficient to induce CFS expression. In the human genome, many regions replicate very late, and in fact replication in more than 1% of the genomic DNA extends to G2 [33], but these late-replicating regions are stable and are not fragile sites. Thus, late replication is an important parameter causing CFS instability but this needs to be combined with other features of CFSs to induce CFS expression.

CFSs have a paucity of replication origins [30, 34]. Mapping of replication initiation events revealed that FRA3B has a shortage of replication origins [35]. Interestingly, this paucity of replication initiation in the FRA3B core extends approximately 700 kilobases in lymphoblastoid cells where FRA3B is unstable, but not in fibroblasts where FRA3B is not expressed [25]. This tissue-specific fragility of FRA3B instability strongly correlates with the shortage of active replication origins. More recently, mapping ORC2 binding sites throughout the human genome found that ORC binds nonspecifically to open chromatin regions containing active marks such as H3 acetylation and H3K4 methylation [23, 24]. There are far more ORC sites in early replicating regions than in late replicating regions, suggesting that ORC density influences replication timing. Large genomic regions with a paucity of ORC sites are strongly associated with CFSs [23, 24], supporting the notion that CFSs often lack of sufficient replication origins. The presence of dormant origins is important for rescuing late and slow replication to complete replication before entering mitosis and thus shortage of origins at CFSs causes insufficient DNA replication at late and slow replication forks in CFS regions especially upon replication stress. Many CFSs co-localize with very large genes, and more than 80% of CFSs in the human genomes contain genes with a size greater than 300 kb [36]. It has been suggested that transcription of human genes larger than 800 kb extends more than one cell cycle. This would inevitably cause transcription and replication collision and induce formation of DNA–RNA hybrids (R loops), which result in DSB formation at CFSs [26]. In another study, it has been shown that large active transcription units (> 1 Mb) are robust cell type-specific predictors of CFS instability and CNV hotspots [36, 37]. Tissue specific CFS expression is largely due to tissue-specific expression of these large genes [26]. Recent study using repli-Seq analysis of the whole genome revealed that more than 80% of replication-delayed regions are transcribed continuously for at least 300 kb, and long-rang transcription removes replication origins out of the gene body, responsible for origin paucity in CFSs containing large genes [38, 39]. However, the expression of large genes does not always correlate with CFS expression [40]. Thus, transcription of large genes is one important contributor to CFS instability but is not a solo player that can sufficiently induce CFS expression.

Sequence analysis of CFSs revealed that CFSs are AT-rich and contain long stretches of interrupted AT-dinucleotides ranging in length from ~ 100 bp to several kilobases [41–44]. More structural study showed that these AT-rich sequences at CFSs (CFS-ATs) exhibit high flexibility and reduced helix stability [45–47]. After DNA unwinding, these CFS-ATs are prone to forming DNA secondary structures such as hairpins, which are more stable than they are in single-strand DNA (ssDNA) configuration. Thus, it is predicted that during DNA replication when CFS-AT sequences are in ssDNA state, secondary structures would form there to stall DNA replication. Indeed, the Freudenreich lab found that such AT-rich sequences derived from FRA16D cause replication fork stalling and chromosomal breakage in yeast [17]. We further showed that multiple CFS-ATs derived from FRA16D and FRA3B cause DSB formation and induce mitotic recombination [48, 49]. An elegant DNA combing analysis from the Kerem group demonstrated that fork arrest at the FRA16C site is preferentially close to the AT-rich sequences [50]. Irony-Tur Sinai, et al. integrated a 3.4 kb AT-rich sequence derived from FRA16C into a stable chromosomal region in the human genome, and showed that this integration drives fragile site formation under conditions of replication stress [16]. Importantly, the recurrent breakpoints found in cancer show significant overlaps with the AT-rich sequences at CFSs [43, 51, 52]. These data strongly suggest that CFS-ATs are one of the important elements contributing to CFS instability. However, like other features of CFSs, forming secondary structures at CFS-ATs per se is not sufficient to commit CFS expression on mitotic chromosomes.

### Replication stress induces CFS instability

CFSs are expressed under conditions that perturb normal DNA replication. CFSs are commonly induced by low concentrations of APH, an inhibitor of DNA polymerases [1]. Folate deficiency and a low dose of hydroxyurea, a ribonucleotide reductase inhibitor, reduce cellular dNTP pools and induce expression of some CFSs [1, 53]. CFS-ATs may form DNA secondary structures on lagging strands during DNA replication, and upon replication stress, more ssDNA is accumulated, leading to increased formation of DNA secondary structures at CFS-ATs, which would further stall DNA replication and cause replication fork collapse. Activation of dormant origins is a checkpoint response to ensure the completion of DNA replication. Since CFSs have a shortage of replication origins, replication often cannot complete at CFSs upon replication stress, leading to CFS expression.

Aberrant oncogene expression induces replication stress [13, 14], which can be mediated by different mechanisms. Oncogene expression prematurely promotes DNA replication and cell proliferation, resulting in an insufficient nucleotide pool [11]. Unscheduled activation of replication origins and an increased number of simultaneous active replication forks upon oncogene expression will also increase conflicts between replication and transcription, resulting in fork collapse [54, 55]. In other cases, expression of oncogenes reduces origin licensing or inhibits origin firing, inducing replication stress [56–59]. Consistent with the notion that replication stress induces CFS instability, oncogene overexpression induces CFS expression [40, 49]. For instance, cyclin E and Ras overexpression in BJ-hTERT cells induces expression of many CFSs, but interestingly, the CFSs that are expressed upon cyclin E and Ras overexpression and APH treatment are partially overlapped but not identical [40]. The underlying mechanism is not clear, but possible different transcription profiles at CFS induced by different oncogenes and APH may cause this difference. It has also been shown that oncogene expression-induced chromosomal instability is predominantly associated with CFSs [11, 13, 14, 60].

Replication checkpoint is involved in maintaining CFS stability. The ATR-mediated replication checkpoint monitors replication progression, functions to protect stalled replication forks, promotes fork restart and coordinates replication and cell cycle progression [61]. Casper and his colleagues showed that ATR, but not ATM, plays a critical role in protecting CFSs; loss of ATR results in CFS expression even in the absence of replication stress [62]. Consistently, depletion of ATR downstream kinase CHK1, but not ATM downstream kinase CHK2, results in CFS expression [63]. Inhibition of checkpoint proteins Claspin and HUS1 also causes CFS expression [64].

### Protection of CFSs to prevent chromosomal breakage

**Fig. 1:**
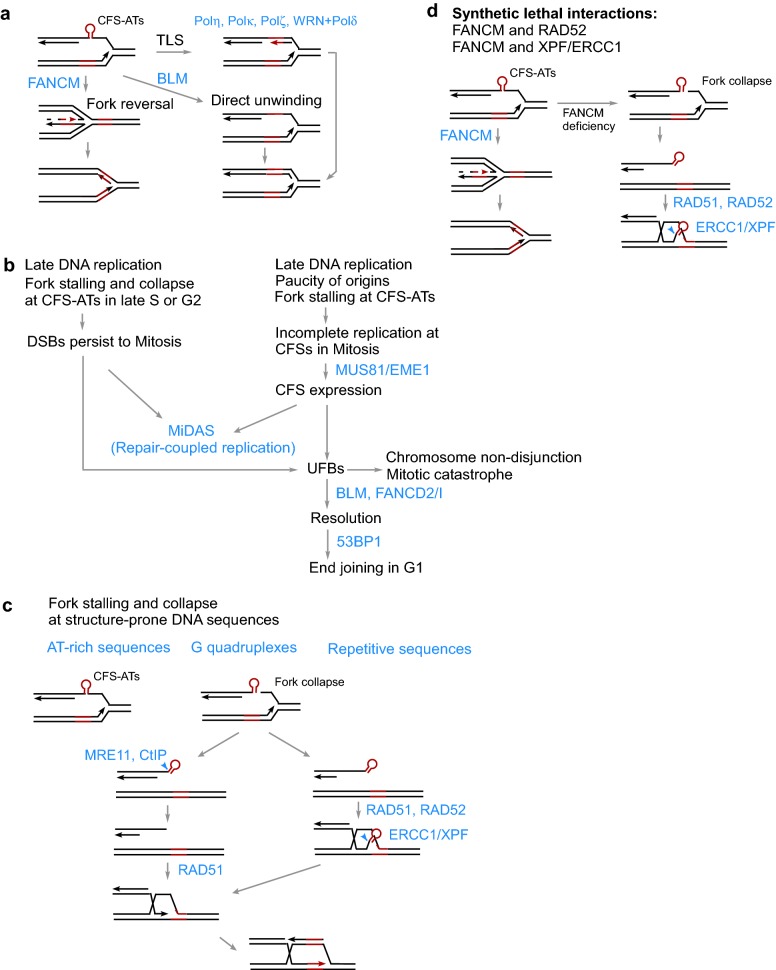
Multiple pathways are involved in protection of CFSs and in repair of DSBs formed at CFSs

Two major mechanisms are used to maintain CFS stability and prevent chromosomal breakages at CFSs during replication. One is to use specialized DNA polymerases via translesion DNA synthesis (TLS) to replicate through structure-forming DNA sequences at CFSs, and the other is to use DNA helicases or translocases to resolve DNA secondary structures when forks are stalled at CFSs (Fig. 1a).

In an in vitro primer extension assay, Pol δ was significantly inhibited in regions containing AT-rich hairpins and microsatellites that are often found in CFSs [19]. Specialized DNA polymerases, such as Pol η, Pol κ and Rev3 are involved in CFS stability maintenance [65, 66]. Both Pol η and Pol κ are able to exchange with Pol δ that is stalled at CFS repetitive sequences, and Pol η and Pol κ are more efficient than Pol δ in replicating non-B form DNA structures in CFSs [19, 65, 67, 68]. In support of the role of Pol η and Pol κ in replicating unusual DNA sequences at CFSs, expression of CFSs is significantly increased in aphidicolin-treated Pol η and Pol κ-deficient cells [65]. Depletion of Rev3 also results in a significant increase in anaphase bridges and CFS expression [66]. WRN deficiency leads to enhanced CFS expression [69]. Interestingly, WRN stimulates in vitro the ability of Pol δ to replicate across DNA secondary structures such as hairpins that are present in fragile sites [70, 71]. Replicating through DNA secondary structures at CFSs alleviates stalling and prevents DSB formation at CFSs.

Multiple helicases and translocases are implicated in resolution of DNA secondary structures formed at CFS-ATs on replication forks. Increased CFS expression was observed in bloom syndrome patients [72]. Our recent studies showed that BLM helicase activity and ATR-mediated phosphorylation of BLM are required for preventing DSB formation at AT-rich sequences in CFSs [73]. Given that BLM efficiently unwinds the bubble and G4 DNA structures in vitro [74], we proposed that BLM is involved in unwinding DNA secondary structures formed at CFS-ATs on replication forks, thereby removing replication blocks and preventing CFS instability. Like BLM, WRN is also considered a DNA structure-specific helicase [74] and is very likely involved in unwinding DNA secondary structures at CFSs.

Fanconi anemia (FA) proteins play an important role in the protection of CFSs [75, 76]. As a component of the FA core complex, FANCM interacts with its binding partners FAAP24 and MHF1/2 (MHF) and is an ATP-dependent DNA-remodeling translocase [77–79]. We showed that FANCM suppresses DSB formation at Flex1 through its translocase activity, which is independent of the FANCI–FANCD2 complex, but requires its binding partners FAAP24 and MHF1/2 [49]. Since FANCM binds specifically to model replication forks and promotes fork reversal in an ATPase-dependent manner in vitro [80, 81], the working model is that upon fork stalling at DNA secondary structures, FANCM activates fork reversal to remove DNA secondary structures and restore normal fork configuration after fork restoration (Fig. 1a). Further study showed that BLM and FANCM are not epistatic to each other [73], supporting the idea that BLM and FANCM use different mechanisms—unwinding DNA secondary structures by BLM and fork reversal by FANCM—to remove DNA secondary structures at CFS-ATs.

It remains to be elucidated how replication bypass and resolution of DNA secondary structures are coordinated with each other to most efficiently promote replication and prevent DSB formation at structure-prone DNA sequences.

### Repair-coupled DNA replication at CFSs in mitosis

Due to late and perturbing DNA replication, along with a shortage of replication origins, under-replicated DNA at CFSs persists into M phase, causing cytogenic manifestation of CFSs as gaps and breaks. In this aspect, MUS81/EME1 and ERCC1 play active roles in cleaving under-replicated DNA at CFSs (Fig. 1b). Although cleavage of under-replicated DNA regions at CFSs in metaphase induces CFS expression, this active cleavage process initiates the DNA replication and repair process, which is important for avoiding anaphase bridge, chromosome mis-segregation and mitotic catastrophes [82, 83]. At the G2 to M transition, MUS81 activity is stimulated upon CDK1-mediated phosphorylation of its partner EME1 [84]. The scaffold protein SLX4, which serves as a binding platform for MUS81/EME1 and ERCC1/XPF [85], is required for recruitment of MUS81/EME1 and ERCC1/XPF to CFSs and promotes controlled DNA processing at CFSs [86]. It has also been found that DNA helicase RECQ5 facilitates CFS cleavage by MUS81-EME1 through removing RAD51 filaments formed on stalled replication forks at CFSs [87].

In a recent study, the Hickson group showed that passage of incompletely replicated DNA at CFSs into mitosis triggers mitotic DNA synthesis (MiDAS) [88]. This MiDAS is increased in cells deficient in Pol η, but requires Pol non-catalytic subunit POLD3 [67, 88], suggesting that break-induced replication (BIR) is used in MiDAS. BIR is a specialized form of HR, which is used when DSBs are single-ended or when homology to the donor is found only at one end of a DSB [89–91]. Interestingly, MUS81/EME1 and SLX4 are required for triggering MiDAS, suggesting that BIR is responsible for completing DNA replication upon cleavage of under-replicated DNA at CFSs by repair-coupled replication in early mitosis [88]. RAD52, which is implicated in BIR in mammalian cells [92], is required for the timely recruitment of MUS81 and POLD3 to CFSs in early mitosis and is important for MiDAS [93]. If DSBs generated at CFSs are not successfully repaired in early mitosis, chromosomal lesions will be transmitted to the daughter G1 cells and are sequestered to specific nuclear bodies that are colocalized with 53BP1 for repair [94, 95].

If under-replicated DNA at CFSs persists into late mitosis, it will lead to the formation of ultrafine anaphase bridges (UFBs), causing chromosome non-disjunction and catastrophe [96]. Depletion of MUS81 or impaired function of BIR in early mitosis results in a significant increase in UFB frequency [88], supporting the notion that endonuclease-mediated specific cleavage of CFSs followed by BIR is critical for completing DNA replication at CFSs. BLM in association with TOPIII/RMI1/RMI2 binds to UFBs and resolves chromosome interlinkage and UFBs for proper chromosomal segregation [97]. FANCD2 and FANCI, which bind to nascent DNA at replication forks, play important roles in regulating origin firing, maintaining fork stability and promoting replication restart [98–101]. Recent study showed that FANCD2 facilitates replication through CFSs in the absence of exogenous stress and does so independently from the Fanconi anemia (FA) core complex and monoubiquitination of FANCD2 [102]. FANCD2 and FANCI also have a role in resolution of UFBs in mitosis. They form mitotic foci at the tips of UFBs, and these foci are dependent on the FA complex proteins which are required for FANCD2 monoubiquitination [96, 103]. It has been shown that FANCD2 and FANCI promote BLM-mediated resolution of UFBs at CFSs, but whether this process requires ubiquitination of FANCD2 has not been investigated yet [96, 103] After BLM-mediated resolution, incompletely replicated DNA regions at CFSs can be rescued by accurate disentanglement of un-replicated DNA, and resulting gaps will be repaired in the following G1 via 53BP1-dependent end joining (Fig. 1b).

### DSB repair at structure-forming DNA sequences upon replication stress

CFSs form cytogenic breaks and gaps at metaphase chromosomes; these are termed late-replicating fragile sites. Genome-wide localization of repair proteins upon replication stress led to identification of early replicating fragile sites (ERFSs) [104]. ERFSs colocalize with highly expressed gene clusters and are enriched for repetitive sequences. Like CFSs, fragility of ERFSs is increased by replication stress and ATR inhibition. The major difference of CFSs and ERFSs is that CFSs often replicate late with gaps and breaks manifested on metaphase chromosomes, while ERFSs replicate early with breaks appeared mainly in S and G2 phases of the cell cycle [29, 104]. In addition, CFSs but not ERFS are enriched with large genes and in short of replication origins. Although ERFSs are GC-rich but CFSs are AT-rich, both tend to form DNA secondary structures when DNA is in ssDNA form, which stalls DNA replication and causes conflict of replication and transcription causing DSB formation. Recent genome-wide mapping of DSB formation sites before and after replication stress revealed that abundant AT-rich sequences are present at spontaneous and replication stress-induced DSB sites, colocalizing with ERFSs, CFSs and ATR inhibition-induced fork collapse sites [105, 106]. These studies suggest that structure-forming DNA sequences are hot spots for chromosome breakage under replication stress.

We showed that AT-rich sequences derived from CFSs induce DSB formation and mitotic recombination both spontaneously and upon replication stress [48, 49]. When CFS-ATs are moved out from native CFSs, they may behave like ERFSs since they form DNA secondary structures and stall DNA replication, which would cause DSB formation in S-phase. However, when in the context of CFS loci, fork collapse at CFS-ATs is expected to occur in late S-phase or even in G2 since CFSs often replicate late, and resulted DSBs may not have sufficient time to be repaired before entering mitosis and cause cytogenic CFS expression at metaphase chromosome (Fig. 1b, left). In addition, fork stalling at AT-rich sequences slows down DNA replication at CFSs and further increases the likelihood of incomplete DNA replication before mitosis at CFSs, which already exhibit the characteristics of late replication initiation and shortage of replication origins (Fig. 1b, right).

Besides AT-rich sequences, other repetitive sequences, microsatellites and G-quadruplexes are also prone to forming DNA secondary structures (Fig. 1c). Similar mechanisms are likely involved in protecting these structure-prone DNA sequences from fork collapse and in repairing DSBs once they are generated there upon fork collapse. We have established EGFP-based DSB reporter systems to analyze the repair of DSBs generated upon fork collapse at CFS-ATs, and these studies can be extended to elucidating DSB repair mechanisms at chromosomal breakage sites carrying other secondary structures. By using an EGFP-based repair reporter, we demonstrated that HR is used as a primary mechanism to repair DSBs caused by fork collapse at CFS-ATs. In addition to their involvement in general end resection, MRE11 and CtIP are specifically required for removing DNA secondary structures present at DSB ends generated at CFS-ATs [107]. ERCC1/XPF is also important for cleaving structure-prone CFS-ATs after DSB formation [108]. Since MRE11 and CtIP are not epistatic to ERCC1/XPF, we propose that MRE11 and CtIP cleave DNA secondary structures before strand invasion while ERCC1/XPF removes these structures after strand invasion (Fig. 1c). Aside from a general requirement for HR proteins, such as BRCA1 and RAD51, to repair DSBs carrying CFS-ATs, intriguingly, RAD52, which is not needed for general HR, becomes indispensable when DSBs contain secondary structures at the ends [49].

Since FANCM plays an important role in removing DNA secondary structures and protecting CFS-ATs, FANCM deficiency causes an accumulation of DNA secondary structures at replication forks, leading to formation of DSBs that contain secondary structures at the ends [49] (Fig. 1c). Since RAD52 and ERCC1/XPF are specifically required for repairing DSBs containing secondary structures, ERCC1/XPF and RAD52 exhibit synthetically lethal interactions with FANCM and are required for FANCM-deficient cells to survive (Fig. 1d) [49, 108]. It remains interesting to test whether defects in other fork protection pathways such as TLS and BLM-mediated unwinding (Fig. 1a) would also cause cell death when RAD52- and ERCC1/XPF-mediated HR pathway is impaired. Although CFS-ATs are limited in numbers, structure-prone DNA sequences are prevalent in our genome. Among DSB sites that have been mapped genome-wide following replication stress, about half (> 30,000 sites) contain AT-rich sequences which are structure-prone [105]. In addition, more than 700,000 sequences are predicted to form G-quadruplexes in the human genome [109]. We anticipate that abundant DNA secondary structures present in the human genome underlie the synthetic lethal interactions of FANCM with ERCC1/XPF and RAD52.

Oncogene overexpression also induces DSB formation and mitotic recombination at CFS-ATs, suggesting that the concerted roles to protect DNA secondary structures are especially important for cancer cell viability. Along these lines, the synthetic lethal interactions of FANCM with ERCC1/XPF and RAD52 provide new strategies for targeted cancer treatment. FANCM is a breast cancer susceptibility gene, and its deficiency has been found in breast tumors, especially triple-negative breast cancer [110–112]. FANCM deficiency has also been described in high grade serous ovarian cancer and sporadic head and neck squamous cell carcinoma [113, 114]. We speculate that inhibition of ERCC1/XPF or RAD52 in FANCM-deficient tumors would effectively eradicate tumor cells with low toxicity to normal cells.

## Conclusion

CFSs are highly associated with chromosomal rearrangement sites in cancers [115]. Establishing a functional link of replication stress and CFS breakage has brought a breakthrough in understanding the mechanisms underlying CFS fragility induced at the early stages of cancer development. Clarifying the roles of various genetic players contributing to CFS instability during tumorigenesis will further advance our understanding of cancer etiology. A large set of observations has revealed the complexity of CFS instability, and among them tissue-specific CFS expression strongly indicates the multifaceted nature of CFS expression. Epigenetic modifications which depend on cell type, cell cycle and the source of replication stress should be taken into accounts to interpret fragility of specific CFSs. Further study of the interplay of different mechanisms and the crosstalk between different pathways would be extremely important for understanding CFS stability and addressing the relevance of CFSs to cancer development and other diseases.

CFSs are defined as cytogenetic chromosomal breakage sites appearing on metaphase chromosomes. Identification of ERFSs and other replication stress sensitive sites by genome-wide analysis revealed a widespread contribution of structure-forming DNA sequences to replication stress-induced chromosomal breakage [105, 106]. These findings strongly support the notion that forming DNA secondary structures is an important contributor not only to CFS instability but also to global genome instability. It also raises interesting questions such as how different structure-forming DNA sequences similarly and differently contribute to genome instability and cancer-related chromosomal rearrangements. Given that structure-forming structures are vulnerable sites for chromosomal breakage in the genome, it is also important to address what the genetic determinants are to distinguish CFSs from other easy-to-break sites in the genome.

Since forming DNA secondary structures appears to be a common mechanism to cause chromosomal breakages spontaneously and upon replication stress, deciphering the mechanisms underlying the protection of these unique DNA sequences becomes an immediate interest for understanding genome stability maintenance. Regarding CFSs, study of damage tolerance and DSB repair mechanisms in the context of other CFS features that cause incomplete DNA replication before entering mitosis will be important for unfolding the complex basis of CFS instability. Study of the genetic interactions of the pathways in protecting CFSs and other structure-forming DNA sequences will also bring new insights into cancer treatment by taking advantage of synthetic lethal interactions of different genetic pathways and the defects or vulnerabilities that are associated with cancers.

## Data Availability

Not applicable.

## Abbreviations

BIR: Break-induced replication
CFSs: Common fragile sites
CNV: Copy number variation
ORC: Origin recognition complex
FA: Fanconi anemia
TLS: Translesion DNA synthesis
UFBs: Ultrafine anaphase bridges
ERFSs: Early replicating fragile sites
MiDAS: Mitotic DNA synthesis
